# Prognostic factors in clinical T1N0M0 thoracic esophageal squamous cell carcinoma invading the muscularis mucosa or submucosa

**DOI:** 10.1186/s13014-016-0660-4

**Published:** 2016-06-21

**Authors:** Yusuke Uchinami, Miyako Myojin, Hiroaki Takahashi, Keiichi Harada, Shinichi Shimizu, Masao Hosokawa

**Affiliations:** Department of Radiation Oncology, Keiyukai Sapporo Hospital, 1-1 Kita, Hondori-14, Shiroishi-ku, Sapporo, 003-0027 Japan; Department of Gastroenterology, Keiyukai Daini Hospital, Hondori-13, Shiroishi-ku, Sapporo, 003-0027 Japan; Department of Radiation Oncology, Hokkaido University Graduate School of Medicine, North-15 West-7, Kita-ku, Sapporo, 060-8638 Japan; Department of Surgery, Keiyukai Sapporo Hospital, Sapporo, Japan

**Keywords:** T1N0M0, Superficial esophageal cancer, Depth of tumor invasion, Chemoradiotherapy, Radiotherapy, Elective nodal irradiation, Endoscopic submucosal dissection, Endoscopic mucosal resection

## Abstract

**Background:**

Multimodality treatment is widely performed for clinical T1N0M0 (UICC-TNM classification, 7th edition) thoracic esophageal squamous cell carcinoma (ESCC), but available articles regarding treatment results are limited. This study assessed the outcomes of clinical T1N0M0 thoracic ESCC invading the muscularis mucosa (MM) or submucosa (SM) treated with radiotherapy (RT) or chemoradiotherapy (CRT).

**Methods:**

We retrospectively reviewed the medical charts of 90 patients with clinical T1N0M0 thoracic ESCC treated with RT or CRT in our hospital in 2004–2011. Of these 90 patients, we analyzed the cases of 71 patients who met our inclusion criteria. All 71 patients had MM or SM cancer. In the 47 patients treated with CRT, the chemotherapy regimen of 5-fluorouracil (5-FU) plus cisplatin (CDDP) was used for 46 patients and 5-FU and nedaplatin was used for one patient. Forty-five patients underwent endoscopic resection (ER) followed by RT or CRT as an additional treatment. Elective nodal irradiation (ENI) was used in 39 patients. For all analyses, statistical significance was defined as 0.05, and the Bonferroni correction was used for the multivariate analysis.

**Results:**

The median age was 70 years (range 47–84). With a median follow-up of 43.6 months (range 1.5–124.2), the 5-year overall survival (OS), disease-specific survival (DSS) and disease-free survival (DFS) rates were 64.0, 72.8 and 50.0 %, respectively. The multivariate analysis showed that performance status (PS) was an independent prognostic factors for DSS and DFS (DSS, *p* < 0.001; DFS, *p* < 0.001). Chemotherapy in addition to RT showed a trend for better DSS (*p* = 0.032) but was not significant following Bonferroni correction. ER and ENI were not significant predictive factors for DSS and DFS.

**Conclusions:**

PS was an independent prognostic factor for DSS and DFS. ER and ENI had no significant relationship with DSS or DFS. The present results may be helpful in treatment decisions for clinical T1N0M0 thoracic ESCC.

**Electronic supplementary material:**

The online version of this article (doi:10.1186/s13014-016-0660-4) contains supplementary material, which is available to authorized users.

## Background

Several therapeutic options have been developed for clinical T1N0M0 (UICC-TNM classification, 7th edition) thoracic esophageal squamous cell carcinoma (ESCC): surgery, radiotherapy (RT), chemoradiotherapy (CRT), endoscopic resection (ER) including endoscopic mucosal resection (EMR), and endoscopic submucosal dissection (ESD). Radical surgery with regional lymph node dissection has been the mainstay of the treatment for clinical T1N0M0 ESCC. Along with the development of endoscopic equipment, ER becomes widely used for treating early-stage esophageal cancer [1, 2]. ER followed by RT or CRT is also one of the treatment strategies for ESCC cases with a higher risk of lymph node or distant metastasis. A retrospective analysis suggested that the overall survival (OS) of patients with superficial ESCC treated by CRT was comparable to that of patients treated with radical surgery [3, 4]. Definitive CRT is another option for ESCC patients who are not candidates for surgery. In their analysis of 72 patients with T1N0M0 ESCC treated by CRT, the Japan Clinical Oncology Group reported that the four-year survival rate was 80.5 % and the four-year major relapse-free survival rate was 68 % [5].

Although clinical T1N0M0 ESCC is categorized as a superficial cancer, the depth of tumor invasion to the mucosal or submucosal layer is closely related to the survival and metastasis rates [6–8]. Based on their retrospective analysis of 402 ESCC patients treated with ER, Yamashina et al. reported that the cumulative 5-year metastasis rates of patients with epithelium/lamina propria (EPM/LPM), muscularis mucosa (MM), and submucosa (SM) cancer were 0.4, 8.7 and 25.7 %, respectively [8]. In UICC-TNM classification (7th edition), T1a includes EPM/LPM and MM cancers, and T1b includes SM cancers. The results of their multivariate analysis indicated that survival and risk of metastasis were associated mainly with invasion into the MM or SM. They concluded that based on the rate of lymph node metastasis, clinical T1N0M0 ESCC invading the MM or SM should be distinguished from EP/LPM cancer.

In organ-conserving treatments for clinical T1N0M0 ESCC, the depth of tumor invasion is closely related to the treatment approach. Additional treatments after ER are essential in MM or SM cancer, due to their higher rates of lymph node metastasis [9]. The indication for additional treatments after ER is based not only on the depth of tumor invasion, but also on pathological findings including the resection margin, venous invasion and lymphatic invasion. In organ-conserving treatments, although some retrospective analyses showed that ER followed by CRT was effective for patients with superficial ESCC [10, 11], the necessity of ER for MM or SM cancer is not yet well understood. The goal of the present study was to determine the prognostic and predictive factors in cases of clinical T1N0M0 thoracic ESCC invading the MM or SM treated with radiotherapy or chemoradiotherapy.

## Methods

### Patients and treatments

We retrospectively reviewed the medical charts of 90 patients with clinical T1N0M0 thoracic ESCC treated with RT or CRT at our hospital between 2004 and 2011. At our hospital, almost all clinical T1N0M0 thoracic ESCC patients are advised to undergo surgery or ER due to its favorable survival rate and safety. However, some patients were not fit for surgery because of complications, their general condition, or age. Therefore, most of the patients in the present series were ineligible for or declined to undergo radical surgery. Of the 90 patients, we identified 71 patients who met all of our inclusion criteria: (1) younger than 85 years of age, (2) histologically proven squamous cell carcinoma, (3) depth of tumor invasion limited to the MM or SM, (4) no lymph node or distant metastasis, (5) no history of esophageal cancer, (6) no other primary cancers at the diagnosis of ESCC, and (7) preserved organ function to undergo definitive RT or CRT. Of the initial 90 patients, 19 patients were excluded: nine patients with synchronous other primary cancers, eight with EP/LPM esophageal cancer, one patient ≥85 years old, and one patient with esophageal adenocarcinoma.

The length and circumferential spread of each tumor was measured for 71 patients (100 %) by esophagogastroduodenoscopy (EGD). The depth of tumor invasion was evaluated for 70 patients (98.6 %) by endoscopic ultrasound (EUS), and the remaining one patients were evaluated comprehensively. For the patients treated with ER before RT or CRT, the depth of tumor invasion was determined by pathological examination. Lymph node or distant metastases were ruled out by EUS and computed tomography (CT) for 71 patients (100 %), and positron emission tomography/computed tomography (PET/CT) for 56 patients (78.9 %). The Eastern Cooperative Oncology Group (ECOG) performance status (PS) was evaluated by physicians or nurses at the diagnosis. Written informed consent was obtained from all patients before each treatment. This retrospective study was approved by the Keiyukai Sapporo Hospital Institutional Review Board.

### Endoscopic resection

Curative ER was performed in 45 patients before their RT or CRT. Of these 45 patients, 42 received ESD and the other three patients received EMR. The ER specimen of each patient was examined pathologically for histological type, depth of tumor invasion, resection margin, lymphatic and venous invasion of tumor.

### Radiotherapy and chemoradiotherapy

RT planning was performed with a CT simulator and radiation treatment planning system: Pinnacle ver. 8.0–9.6 (Philips, Eindhoven, The Netherlands). We defined the regional lymph node area as the bilateral supraclavicular, periesophageal, mediastinal and perigastric lymph node areas. We defined the prophylactic RT that included these lymph node areas as elective nodal irradiation (ENI). For primary tumors that did not undergo ER, the gross tumor volume (GTV) was contoured on the planning CT images according to all available resources. The GTV was expanded to the clinical tumor volume (CTV) by extending a 2–3-cm margin superiorly and inferiorly, and a 0.5-cm margin laterally. In the ENI cases, the CTV included all prophylactic regional lymph nodes together with the primary tumor or tumor bed. In the non-ENI cases, the CTV included the primary tumor or tumor bed with an optional part of the regional lymph nodes. The planning target volume (PTV) was then created by adding a 1–1.5-cm margin to the CTV.

The techniques of ENI were different during the periods when RT was performed. From July 2004 to March 2009, the dose of 39.6–48 Gy in 20–24 fractions was delivered to the isocenters with anterior/posterior opposed portals. From April 2009 to 2011, the dose of 50.4 Gy in 28 fractions was delivered with anterior/posterior opposed and additional oblique portals [12]. In non-ENI, the dose of 39.6–48 Gy in 20–24 fractions was delivered with anterior/posterior opposed portals. After the initial plan was completed, a tumor boost of 9–20 Gy was delivered with cord-sparing oblique portals to the primary tumor or tumor bed after ER. All patients were treated 5 days a week.

The standard dose of chemotherapy was 5-fluorouracil (5-FU) 700 mg/m^2^ on days 1–5 and cisplatin (CDDP) 70 mg/m^2^ on day 1. Two courses of 5-FU plus CDDP (FP) were administered during radiotherapy at intervals of at least 3 weeks depending on the hematological data or the general condition of the patient. For one patient with decreased renal function, nedaplatin (CDGP) was used instead of CDDP. A reduced dose (75 % of the standard dose) was used for the patients who were ≥80 years old and the patients with a limited daily activity level.

### Follow-up

After their RT or CRT was completed, all patients were included in the follow-up program. The EGD and CT were performed every 4–6 months for the first 5 years. After 2006, when a small lesion was observed on CT images at a periodic follow-up, PET-CT was also applied to determine whether or not the small lesion was metastatic. When suspected lesions were found by EGD, biopsies of the esophagus were also performed. Lymph node or distant metastases were examined by CT or PET-CT. Lymph node metastasis was recorded according to the Japanese Society for Esophageal Diseases’ classification of esophageal cancer, 10th edition [13]. The patients who had been diagnosed with local recurrence or metastasis received salvage treatment including ER, surgical operation, chemotherapy, RT, CRT or palliative treatment.

### Statistical analysis

The follow-up started on the first day of radiotherapy and ended as one of the following: date of death, date of the patient’s last visit to our hospital, or date that the patient was last known to be alive confirmed by telephone interview or a letter from the patient’s referring physician. We calculated the OS rate and the disease-specific survival (DSS) rate from the start date of RT to the date of the last follow-up. We calculated the disease-free survival (DFS) rate from the start date of RT to the earliest occurrence of recurrences or death. Residual tumors after RT or CRT were counted as treatment failure immediately.

Toxicities were scored according to the National Cancer Institute Common Terminology Criteria for Adverse Events (NCI-CTCAE) version 4.0. We used the Kaplan-Meier method to determine the survival rates, with statistical significance assessed by the log-rank test. We used Cox proportional hazards models to evaluate potential associations between the clinical factors and DSS or DFS. For all analyses, a two-sided *p*-value <0.05 was considered significant. For the multivariate analysis, the Bonferroni correction was used and the significance value of 0.05 was divided by the number of factors to obtain an adjusted significance level. The statistical analyses were conducted using SPSS software (version 23; IBM SPSS statistics, Chicago, IL, USA).

## Results

### Patient characteristics and treatments

The patients’ characteristics are summarized in Table 1. Of all 71 patients, 57 patients were PS 0, ten were PS 1 and four were PS 2 or worse. The reasons of three PS 3 cases were as follows: vertebral compression fracture, lower body paralysis after a traffic accident and left hemiparalysis after a cerebral infarction. One patient was PS 2 because of old age. Of all 71 patients, 18 patients underwent RT, eight were administered CRT, six underwent ER followed by RT, and 39 underwent ER followed by CRT. Of the 45 patients treated with ER, 42 patients underwent ESD and three underwent EMR. Two patients were unable to finish the prescribed radiation course; RT of one patient was stopped at the dose of 28 Gy due to radiation pneumonitis, and the other patient’s RT was stopped at the dose of 52 Gy for a private reason. In the RT group, 20 of the 24 patients were treated with a local RT field, whereas in the CRT group, 35 of the 47 patients were treated with ENI. Of the 47 patients in the CRT group, 35 were treated with two cycles of standard-dose FP, six patients were treated with two cycles of low-dose FP, and the other six patients received one cycle of FP or CDGP instead of CDDP.

**Table 1:** Patient characteristics and treatments

	Number	Percent
Patients	71	
Age (years)		
Median	70	(range 47–84)
Sex		
Male	57	80.3
Female	14	19.7
Performance status		
0	57	80.3
≥ 1	14	19.7
Observation period (months)		
Median	43.6	(range 1.5–124.2)
Main tumor location		
Upper thoracic	12	16.9
Middle thoracic	41	57.7
Lower thoracic	18	25.4
Depth of tumor invasion		
Muscularis mucosa	6	8.5
Submucosa	65	91.5
Tumor length (cm)		
Median	4.0	(range 1–30)
Circumferential spread of tumor		
Median	0.50	(range 0.17–1)
Tumor number		
1	65	91.5
≥ 2	6	8.5
Treatment		
RT	18	25.4
CRT	8	11.3
ER + RT (ESD/EMR)	6 (4/2)	8.5
ER + CRT (ESD/EMR)	39 (38/1)	54.9
RT field		
Non-ENI	32	45.1
ENI	39	55.0
Radiation dose (Gy)		
Median	50.4	(range 28–68)
Chemotherapy		
Standard dose FP (2 cycles)	35	74.5
Low-dose FP (2 cycles)	6	12.8
Others	6	12.8

*CRT* chemoradiotherapy, *EMR* endoscopic mucosal resection, *ENI* elective nodal irradiation, *ER* endoscopic resection, *ESD* endoscopic submucosal dissection, *FP* 5FU + Cisplatin, *RT* radiotherapy

### Disease-specific survival

With a median follow-up of 43.6 months, the 5-year OS and DSS rates of all 71 patients were 64.0 and 72.8 %, respectively. Of all cases, 15 patients died due to their ESCC or a treatment-related event. Five patients died due to the following (one patient each): suicide, cerebral infarction, lymphoma, myocardial infarction, and heart failure.

**Fig. 1:**
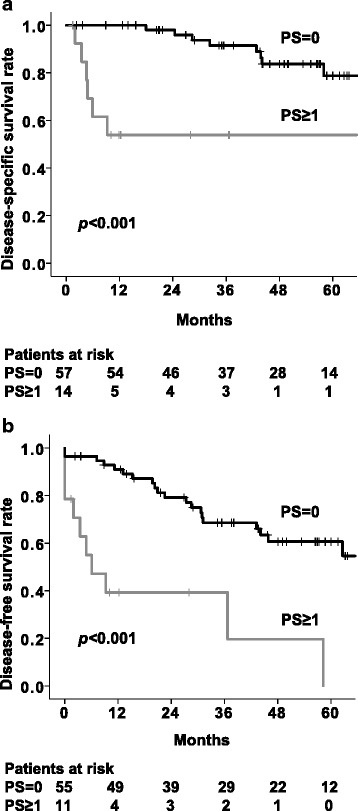
Disease-specific survival **a** and disease-free survival **b** rates in the PS 0 and PS 1–3 patients

Thus, to identify ESCC-related predictive and prognostic factors, we used univariate and multivariate analyses for DSS. In the univariate analysis, PS (=0), radiation dose (≤50 Gy), ER, and CRT were significant predictive and prognostic factors (Table 2). The multivariate analysis revealed that PS and CRT were significant factors, with hazard ratios (HRs) of 8.72 (95 % confidence interval [CI]: 2.76–27.59, *p* < 0.001) for PS and 0.29 (95%CI: 0.092–0.90, *p* = 0.032) for CRT (Table 3). After adjustment with the Bonferroni correction, PS was the only significant predictive factor. For the patients with PS 0 and those with PS 1–3, the 5-year DSS rates were 78.8 and 53.8 % (*p* < 0.001), respectively (Fig. 1a).

**Table 2:** Univariate analysis for disease-specific survival and disease-free survival rates

	n	5-year DSS (%)	*p*-value	5-year DFS (%)	*p*-value
Age					
≤ 70	37	77.8	0.574	55.4	0.117
> 70	34	65.7		44.3	
Sex					
Male	57	70.9	0.925	49..7	0.917
Female	14	76.2		51.9	
Performance status					
0	57	78.8	<0.001	60.7	<0.001
≥ 1	14	53.8		0	
Main tumor location					
Upper thoracic	12	0	0.205	64.8	0.186
Middle thoracic	41	80.1		57.2	
Lower thoracic	18	74.9		25.5	
RT field					
Non-ENI	32	62.3	0.094	42.0	0.389
ENI	39	81.7		55.3	
Radiation dose					
≤ 50 Gy	32	88.5	0.003	61.6	0.035
> 50 Gy	39	55.0		38.0	
Tumor length					
≤ 5 cm	55	70.5	0.540	50.9	0.818
> 5 cm	16	85.2		50.6	
Circumferential spread of tumor					
≤ 0.75	63	71.2	0.464	47.7	0.302
> 0.75, ≤1	8	83.3		68.6	
Tumor number					
1	65	62.8	0.170	51.2	0.149
≥ 2	6	100		44.4	
Depth of tumor invasion					
Muscularis mucosa	6	100	0.227	26.7	0.808
Submucosa	65	70.7		51.3	
Endoscopic resection					
No	26	53.6	0.005	33.8	0.014
Yes	45	82.9		58.8	
Chemotherapy					
No	24	54.5	0.005	44.1	0.036
Yes	47	80.8		53.7	

*DSS* disease-specific survival, *DFS* disease-free survival, *ENI* elective nodal irradiation, *RT* radiotherapy

**Table 3:** Multivariate analysis for disease-specific survival and disease-free survival rates

	Disease-specific survival		Disease-free survival	
Characteristics	HR (95 % CI)	*p*-value	HR (95 % CI)	*p*-value
Performance status				
0	1	<0.001*	1	<0.001*
≥ 1	8.72 (2.76–27.59)		5.31 (2.40–11.78)	
Radiation dose				
≤ 50 Gy	1		1	0.618
> 50 Gy	2.15 (0.46–10.07)	0.331	1.31 (0.45–3.81)	
Endoscopic resection				
No	1	0.268	1	0.656
Yes	2.67 (0.47–15.20)		1.31 (0.40–4.30)	
Chemotherapy				
No	1	0.032	1	0.184
Yes	0.29 (0.092–0.90)		0.59 (0.27–1.29)	

*HR* hazard ratio, *CI* confidential interval, *ENI* elective nodal irradiation, *RT* radiotherapy. *Indicates significance after adjustment with Bonferroni correction (*p*-value < 0.0125)

### Disease-free survival and patterns of recurrences

Recurrences were observed in 23 patients, including nine with local recurrence, nine with regional lymph node metastasis, five with distant metastasis. After their recurrences were found, seven patients underwent salvage ER, nine received chemotherapy, three underwent surgery and the remaining four patients received other treatments. Of the 23 patients with recurrences, 13 had undergone CRT for their primary ESCC (Table 4). In these patients, lymph node metastases tend to be found more often in the right neck or right upper mediastinum than other sites.

**Table 4:** Recurrences in patients treated with chemoradiotherapy

PS	Location	ER	RT field	Dose	Recurrence site	DFS	Treatment for recurrence
0	Mt	−	ENI	60.0	Local	0	Surgery
1	Mt	+	ENI	28.0	Lung	6.1	Surgery
0	Ut	−	Non-ENI	40.0	Local	7.2	ESD
0	Mt	+	ENI	40.0	#104R	13.1	Chemotherapy
0	Lt	+	ENI	39.6	#3, #7	15.3	Surgery
0	Ut	+	ENI	50.4	#101R	19.8	Chemotherapy
0	Mt	+	ENI	40.0	#109R	22.5	Chemotherapy
0	Mt	+	ENI	39.6	#104R	28.5	Chemotherapy
0	Mt	+	ENI	65.0	#104R	30.7	Chemotherapy
0	Mt	+	Non-ENI	40.0	#104R	31.0	Chemotherapy
0	Mt	+	ENI	39.6	Local	43.2	ESD
1	Lt	−	Non-ENI	65.0	#106recR	58.3	Chemotherapy
0	Mt	+	ENI	40.0	Local	75.1	ESD

Lymph node metastasis was recorded according to the Japanese Society for Esophageal Diseases classification of esophageal cancer, 10th edition. #104: supraclavicular LNs (lymph nodes), #3: LNs along the lesser curvature, #7: LNs along the left gastric artery, #101: cervical paraesophageal LNs, #109: main bronchus LNs, #106rec: recurrent nerve LNs. *DFS* disease-free survival (months), *Dose* radiotherapy dose (Gy), *ER* endoscopic resection, *ESD* endoscopic submucosal dissection, *Lt* lower thoracic esophagus, *Mt* middle thoracic esophagus, *PS* Performance status, *RT field* radiotherapy field, *Ut* upper thoracic esophagus

The 5-year DFS rate of all 71 patients was 50.0 %. The univariate analysis for each clinical factor identified PS (=0), radiation dose (≤50 Gy), ER, and CRT as predictive factors for DFS (Table 3). The multivariate analysis showed that PS was an independent prognostic factor for DFS with the HR of 5.31 (95%CI: 2.40–11.78, *p* < 0.001). The 5-year local control rate was 86.6 %, and ER was shown by the multivariate analysis to be a significant predictive factor (Additional file 1: Table S1 and Additional file 2: Table S2). In the patients with PS 0 and PS 1–3, the 5-year DFS rates were 60.7 and 0 % (*p* < 0.001), respectively (Fig. 1b).

### Late toxicity

Adverse late events (≥ grade3) were identified in five patients, all of whom had been administered CRT (Table 5). Radiation pneumonitis developed in two patients. Of these two patients, one patient had refused medical treatment for radiation pneumonitis and died 20 days after CRT. The 60-year-old patient with grade 5 toxicity had had liver cirrhosis before CRT and died due to heart failure at 43 months after CRT. The other grade 5 toxicity case, a 76-year-old patient, had had hypertension and died due to myocardial infarction at 45 months after CRT.

**Table 5:** Late toxicities in the 71 patients

Adverse events	Grade 3 or 4	Grade 5
Radiation pneumonitis	1	1
Pleural effusion	1	0
Myocardial infarction	0	1
Heart failure	0	1

Toxicities were scored according to the NCI-CTCAE ver. 4.0

## Discussion

The present analysis of organ-conserving treatment for patients with clinical T1N0M0 thoracic ESCC demonstrated that PS was an independent prognostic factor for both DSS and DFS. ER was not a significant predictive factor in the multivariate analysis despite its significance in local control. Although a few research groups suggested that the combined treatment of ER followed by CRT was effective [10, 11], the role of ER in MM or SM esophageal cancer is controversial. The present results may be helpful for choosing the appropriate treatment and predicting the prognosis for each patient.

Our multivariate analysis with Bonferroni correction revealed that CRT was not an independent predictive factor for DSS and DFS. For DSS, however, CRT showed a trend for better survival (*p* = 0.032 without Bonferroni correction). Few articles reported significant effectiveness of CRT compared to RT alone in superficial ESCC. Cooper et al. reported that CRT resulted in increased OS rate compared to RT alone, but their trial included locally advanced squamous cell carcinoma and adenocarcinoma cases [14]. In their analysis of 464 surgically resected cases after EMR, Eguchi et al. reported that ESCC invading the MM or SM was a high-risk condition for lymph node metastasis [9]. They proposed that MM or SM cancer should be distinguished from EP or LPM cancer and treated as a systemic disease due to their higher potential for metastasis.

In our present retrospective analysis of 71 patients with MM or SM cancer, 47 patients were treated with CRT. Of these 47 patients, 13 patients (27.7 %) were diagnosed with tumor recurrence. Lymph node or distant metastasis was identified in 9 of these 13 patients during the follow-up period. Despite the early clinical stage, the ESCC invading the MM or SM cancer easily metastasized. These patients might have had occult metastasis at the time of diagnosis. Because ER is a local treatment for MM or SM lesions, chemotherapy with RT may be effective for eliminating these microscopic metastases. In the present study, three patients with grade 5 toxicity had undergone CRT. The death of one of these three patients, a 71-year-old patient with radiation pneumonitis, was speculated to be directly associated with treatment toxicity, although the patient had refused to undergo necessary treatments. For the other two patients with grade 5 toxicity, it is difficult to determine whether the CRT was directly associated with their death considering their ages and medical histories. However, there is no doubt that CRT is a more invasive treatment than RT alone. Therefore, the treatment indications of CRT should be decided carefully depending on the patients’ backgrounds. The present results may be helpful in making treatment decisions for clinical T1N0M0 thoracic ESCC.

Our multivariate analysis showed that PS was the only independent prognostic factor for DSS and DFS. Our study included 14 patients who were PS1–3 because they were ineligible or refused to undergo radical surgery. These patients’ backgrounds might have contributed to the significance of PS. Previous studies have reported that PS was a prognostic factor for survival in patients with stage I–IV esophageal cancer treated with RT or CRT [15–17].

In MM or SM esophageal cancer treated with definitive RT or CRT, the effectiveness of ER is still controversial. According to the Japan Esophageal Society guidelines for the treatment of esophageal cancer, the absolute indication for ER is EP or LPM cancer [18]. Lesions reaching the MM or SM are defined as relative or investigational indications because these cancers have a risk of lymph node or distant metastasis. Shimizu et al. reported that ER combined with CRT was favorable treatment for patients with ESCC invading the MM or upper SM, because none of 15 patients treated with ER followed by CRT had local recurrence or metastasis [10]. However, the statistical analysis in their article was insufficient because of the small number of patients.

Based on their retrospective analysis of patients with ESCC invading the MM or SM, Kawaguchi et al. also reported that patients treated with ESD followed by CRT had favorable OS compared to the patients treated with CRT alone [11]. Although their paper described the effectiveness of ER followed by CRT for patients with superficial ESCC, no significant difference was found in the survival rate between patients treated with ER and without ER. Because their study included superficial ESCC patients with lymph node metastasis, the staging variability in the enrolled patients might have affected the treatment outcomes.

To investigate the role of ER in superficial ESCC treated with RT or CRT, the accurate diagnosis of the depth of tumor invasion is critical. In superficial ESCC treated with ER, this treatment not only allows us to determine the precise depth of tumor invasion; it is linked to other prognostic factors such as the histological type, the lymphatic and vascular involvement. In superficial ESCC, EUS is considered to be most useful imaging technique for evaluating the depth of tumor invasion, and its diagnostic accuracy for mucosal and submucosal cancers has been reported to be satisfactory [19, 20]. In our present series of 71 patients, EUS was performed in 70 patients (98.6 %), and this high rate of EUS use supports its credible diagnosis of the depth of tumor invasion. In our multivariate analysis for patients with clinical T1N0M0 thoracic ESCC invading the MM or SM, ER showed significance regarding the local control rate. However, ER was not shown to be an independent predictive factor for DSS or DFS. ER is useful for local control and is the definitive method to identify the depth of tumor invasion; however, this result suggests that aggressive ER for MM or SM cancers, which need additional treatment, is not necessarily beneficial for survival.

ENI was not an independent predictive factor for DSS or DFS in our study. Regarding the RT field, there is no definitive consensus regarding whether or not ENI is effective. Yamashita et al. retrospectively analyzed 126 patients with stage I–IV thoracic ESCC treated with CRT, and they concluded that ENI was effective for preventing regional lymph nodal failure, because no patient experienced elective nodal failure without having any other site of recurrence [21]. In their analysis of 102 stage I–IV ESCC patients treated with CRT, Onozawa et al. also reported that ENI was effective for preventing regional nodal failure, because only one patient (1.0 %; 95 %CI, 0–5.3 %) experienced elective nodal failure without any other site of recurrence after achieving complete remission [22]. In contrast, Zhao et al. analyzed 53 patients with T1N0–T4N1 ESCC without distant metastases treated with RT, and they concluded that the omission of ENI was not associated with a significant amount of failure in lymph node regions [23]. One of the challenges in these studies is that they included ESCC cases at various stages. Moreover, chemotherapy may have contributed to the prevention of lymph node metastasis in the previous studies. As our research shows, ENI may not necessarily be required for early ESCC.

A limitation of our study is that it was a non-randomized and retrospective analysis of a single institution’s data. The treatment strategies for each patient also differed somewhat over time. Some of the strengths of our study include the relatively large number of patients, accurate staging using PET-CT or EUS, and the long follow-up period. To identify predictive and prognostic factors in clinical T1N0M0 ESCC, a prospective randomized controlled study of a large number of patients is required.

## Conclusion

The results of this study showed that PS was an independent prognostic factor for both DSS and DFS in patients with clinical T1N0M0 thoracic ESCC invading the MM or SM. CRT showed a trend for better DSS (*p* = 0.032) but was not significant after Bonferroni correction. ER and ENI were not identified as significant predictive factors.

## Abbreviations

3D-CRT, three-dimensional conformal radiotherapy; 5-FU, 5-fluorouracil; CDDP, cisplatin; CRT, chemoradiotherapy; CTCAE, National cancer institute common toxicity criteria; CTV, clinical target volume; DFS, disease-free survival; DSS, disease-specific survival; EGD, esophagogastroduodenoscopy; EMR, endoscopic mucosal resection; ENI, elective nodal irradiation; EPM, epithelium; ER, endoscopic resection; ESCC, esophageal squamous cell carcinoma; ESD, endoscopic submucosal resection; EUS, endoscopic ultrasound; FP, 5-fluorouracil plus cisplatin; GTV, gross tumor volume; LPM, lamina propria; MM, muscularis mucosa; OS, overall survival; PET, positron emission tomography; PTV, planning target volume; RT, radiotherapy; SM, submucosa.

## Additional files

Additional file 1: Table S1. Univariate analysis for local control rate. (DOCX 14 kb) Additional file 2: Table S2. Multivariate analysis for local control rate. (DOC 29 kb)
