# Supplementary material for: Prognostic factors in clinical T1N0M0 thoracic esophageal squamous cell carcinoma invading the muscularis mucosa or submucosa
Source: Radiat Oncol. 2016 Jun 21;11:84. doi: 10.1186/s13014-016-0660-4 (PMC4915080; doi:10.1186/s13014-016-0660-4)
Supplement: Additional file 1: Table S1. — Univariate analysis for local control rate. (DOCX 14 kb) [file 13014_2016_660_MOESM1_ESM.docx]

**Additional file 1: Table S1.** Univariate analysis for local control rate.

|  | **n** | **5-year**  **LC (%)** | ***p*-value** |
| --- | --- | --- | --- |
| Age |  |  |  |
| ≤70 | 37 | 90.0 | 0.257 |
| >70 | 34 | 83.0 |  |
| Sex |  |  |  |
| Male | 57 | 92.3 | 0.461 |
| Female | 14 | 71.3 |  |
| Performance status |  |  |  |
| 0 | 57 | 86.9 | 0.735 |
| ≥1 | 14 | 92.9 |  |
| Main tumor location |  |  |  |
| Upper thoracic | 12 | 90.0 | 0.788 |
| Middle thoracic | 41 | 83.7 |  |
| Lower thoracic | 18 | 94.4 |  |
| RT field |  |  |  |
| Non-ENI | 32 | 79.8 | 0.357 |
| ENI | 39 | 90.1 |  |
| Radiation dose |  |  |  |
| ≤50 Gy | 32 | 94.7 | 0.022 |
| >50 Gy | 39 | 78.4 |  |
| Tumor length |  |  |  |
| ≤5 cm | 55 | 89.6 | 0.249 |
| >5 cm | 16 | 77.8 |  |
| Circumferential spread of tumor |  |  |  |
| ≤0.75 | 63 | 84.5 | 0.272 |
| >0.75, ≤1 | 8 | 100 |  |
| Tumor number |  |  |  |
| 1 | 65 | 87.6 | 0.294 |
| ≥2 | 6 | 66.7 |  |
| Depth of tumor invasion |  |  |  |
| Muscularis mucosa | 6 | 100 | 0.242 |
| Submucosa | 65 | 85.5 |  |
| Endoscopic resection |  |  |  |
| No | 25 | 64.7 | <0.001 |
| Yes | 46 | 95.7 |  |
| Chemotherapy |  |  |  |
| No | 24 | 72.0 | 0.020 |
| Yes | 47 | 91.6 |  |

LC: local control, ENI: elective nodal irradiation, DFS: disease-free survival, RT: radiotherapy.
