# Supplementary material for: Prognostic factors in clinical T1N0M0 thoracic esophageal squamous cell carcinoma invading the muscularis mucosa or submucosa
Source: Radiat Oncol. 2016 Jun 21;11:84. doi: 10.1186/s13014-016-0660-4 (PMC4915080; doi:10.1186/s13014-016-0660-4)
Supplement: Additional file 2: Table S2. — Multivariate analysis for local control rate. (DOC 29 kb) [file 13014_2016_660_MOESM2_ESM.doc]

**Additional file 2: Table S2.** Multivariate analysis for local control rate

|  | **Local control rate** | |
| --- | --- | --- |
| Characteristics | **HR (95%CI)** | ***p*-value** |
| Endoscopic resection |  |  |
| No | 1 | 0.005* |
| Yes | 0.051 (0.006–0.42) |  |
| Chemotherapy |  |  |
| No | 1 | 0.946 |
| Yes | 1.06 (0.21–5.32) |  |

HR: hazard ratio, CI: confidential interval, ENI: elective nodal irradiation, RT: radiotherapy. *Indicates significance after adjustment with Bonferroni correction (p-value<0.025)
